# Kalirin and CHD7: novel endothelial dysfunction indicators in circulating extracellular vesicles from hypertensive patients with albuminuria

**DOI:** 10.18632/oncotarget.14948

**Published:** 2017-02-01

**Authors:** Fernando de la Cuesta, Montserrat Baldan-Martin, Rafael Moreno-Luna, Gloria Alvarez-Llamas, Laura Gonzalez-Calero, Laura Mourino-Alvarez, Tamara Sastre-Oliva, Juan A. López, Jesús Vázquez, Gema Ruiz-Hurtado, Julian Segura, Fernando Vivanco, Luis M. Ruilope, Maria G. Barderas

**Affiliations:** ^1^ Department of Vascular Physiopathology, Hospital Nacional de Paraplejicos (HNP), SESCAM, Toledo, Spain; ^2^ Department of Immunology, IIS-Fundacion Jimenez Diaz, Madrid, Spain; ^3^ Unidad de Proteomica CNIC, Madrid, Spain; ^4^ Unidad de Hipertension, Instituto de Investigacion i + 12, Hospital Universitario 12 de Octubre, Madrid, Spain; ^5^ Departamento de Bioquimica y Biologia Molecular I, Universidad Complutense, Madrid, Spain; ^6^ Current address: Centre for Cardiovascular Science, Queen's Medical Research Institute, University of Edinburgh, Edinburgh, UK

**Keywords:** extracellular vesicles, proteomics, endothelial dysfunction, hypertension, albuminuria

## Abstract

Despite of the great advances in anti-hypertensive therapies, many patients under Renin-Angiotensin- System (RAS) suppression develop albuminuria, which is a clear indicator of therapeutic inefficiency. Hence, indicators of vascular function are needed to assess patients’ condition and help deciding future therapies.

Proteomic analysis of circulating extracellular vesicles (EVs) showed two proteins, kalirin and chromodomain-helicase-DNA-binding protein 7 (CHD7), increased in albuminuric patients. A positive correlation of both with the expression of the endothelial activation marker E-selectin was found in EVs. *In vitro* analysis using TNFα-treated adult human endothelial cells proved their involvement in endothelial cell activation.

Hence, we propose protein levels of kalirin and CHD7 in circulating EVs as novel endothelial dysfunction markers to monitor vascular condition in hypertensive patients with albuminuria.

## INTRODUCTION

Albuminuria has been associated with increased cardiovascular (CV) risk and allows monitoring therapeutic efficiency in hypertensive patients, since a substantial number of them develop de novo albuminuria despite of the RAS suppression treatment [1]. These patients show a characteristic inflammatory signature in both plasma [2, 3] and urine [4]. In this context, increased shear stress, endothelial dysfunction [5] and oxidative stress [6] in the whole circulatory system arises and therefore additional therapies need to be addressed to prevent future events and avoid organ damage.

EVs are membrane vesicles released by secreting cells to communicate with other cells. These vesicles are a very useful tool to understand the processes taking place in the secreting cell, especially in pathological conditions, in which its release is enhanced [7]. EVs include two different types of vesicles: 1) microvesicles (MVs), 50 nm-1 μm vesicles which bud directly from the plasma membrane; and 2) exosomes: 50–120 nm vesicles released by fusion of multivesicular endosomes (MVEs) to the plasma membrane [8]. EVs derived by either platelets (PEVs) or endothelial cells (EEVs) are shed to the bloodstream and increased number of both has been associated with increased CV risk [9]. Furthermore, an increased number of apoptotic EEVs has been recently reported in hypertensive patients with albuminuria [10] and increased shear stress might be the major cause of such increase.

Circulating EVs may therefore constitute a low-invasive tool for analysing the pathogenic processes triggered by albuminuria. Using differential proteomic analysis we have been able to identify kalirin and CHD7 to be increased in EVs from albuminuric patients. *In vitro* analysis showed and proved their involvement in endothelial cell (EC) activation within blood vessels. Thus, we propose protein levels of kalirin and CHD7 in circulating EVs as novel endothelial dysfunction markers in hypertensive patients with albuminuria.

## RESULTS

### Efficient isolation of EVs from blood of hypertensive patients

**Figure 1:**
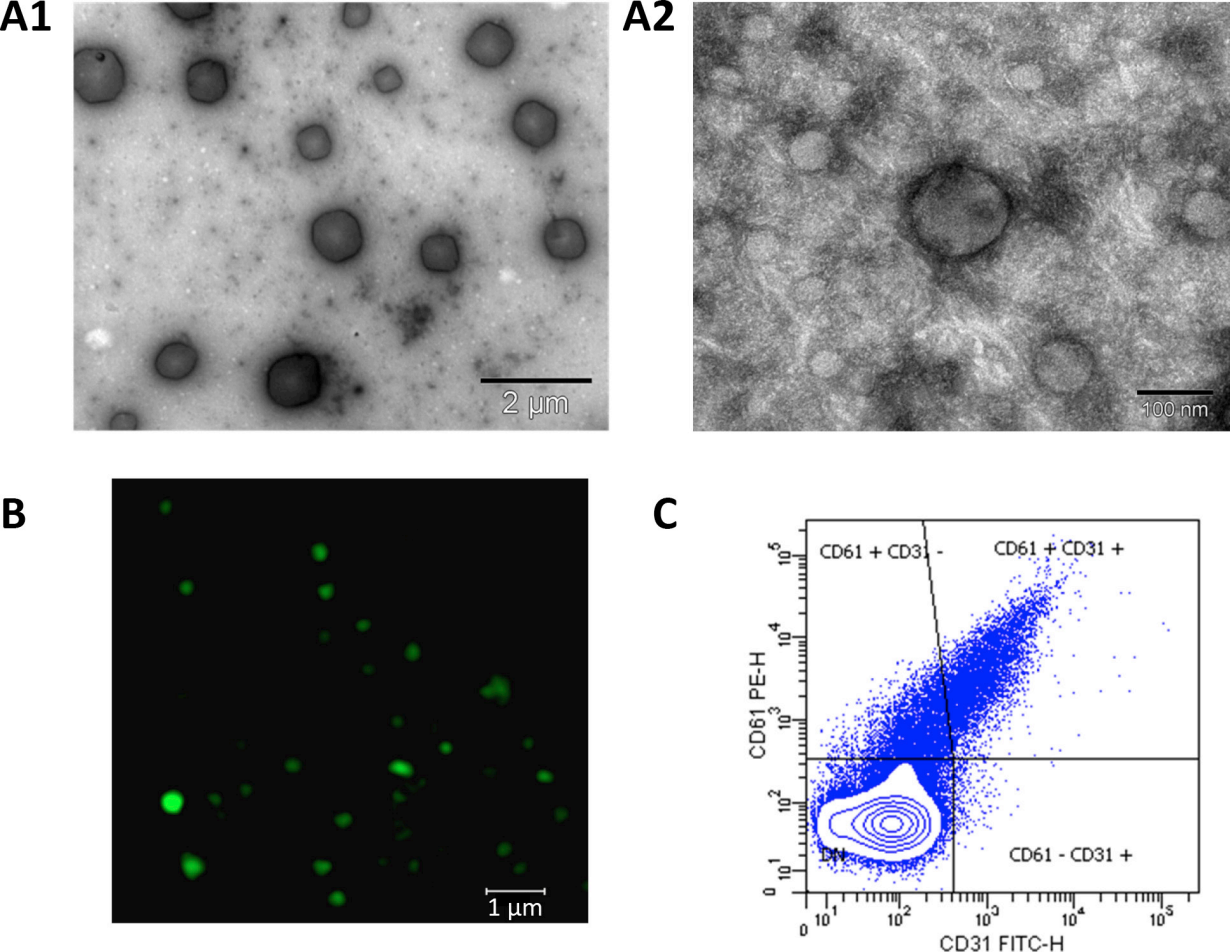
Isolation of EVs from blood plasma of hypertensive patients Electron microscopy allowed to check presence of all range of EVs: (**A1**), (15,000× magnification, scale bar 2 μm) vesicles of 0.1–1 μm size (microvesicles, MVs). (**A2**), (200,000× magnification, scale bar 100 nm) vesicles of 40–100 nm, corresponding to exosomes as well as a small MV in the centre. (**B**) Confocal microscopy analysis of CD61 allowed checking for the presence of platelet-derived MVs, which are the most abundant in the blood (63× oil immersion objective, 1.4 zoom. Scale bar 1 μm). (**C**) Flow cytometry analysis was performed after defining a gate for EVs using Megamix beads. EVs expressing CD61 (platelet-derived) and CD61^−^/CD31^+^(endothelial-derived) were detected in the isolated fraction.

The EV fraction obtained after isolation by ultracentrifugation was checked by Electron Microscopy (EM) confocal microscopy and flow cytometry. EM results showed presence of vesicles ranging from 50 nm – 1 μm size, which corresponded to both exosomes and MVs (Figure 1, A1 and A2). Most abundant CD61^+^ PEVs were imaged by confocal microscopy (Figure 1B) and these, together with CD61^−^/CD31^+^ EMVs were detected by flow cytometry (Figure 1C).

### Differential analysis of EVs from hypertensive patients with albuminuria

**Figure 2:**
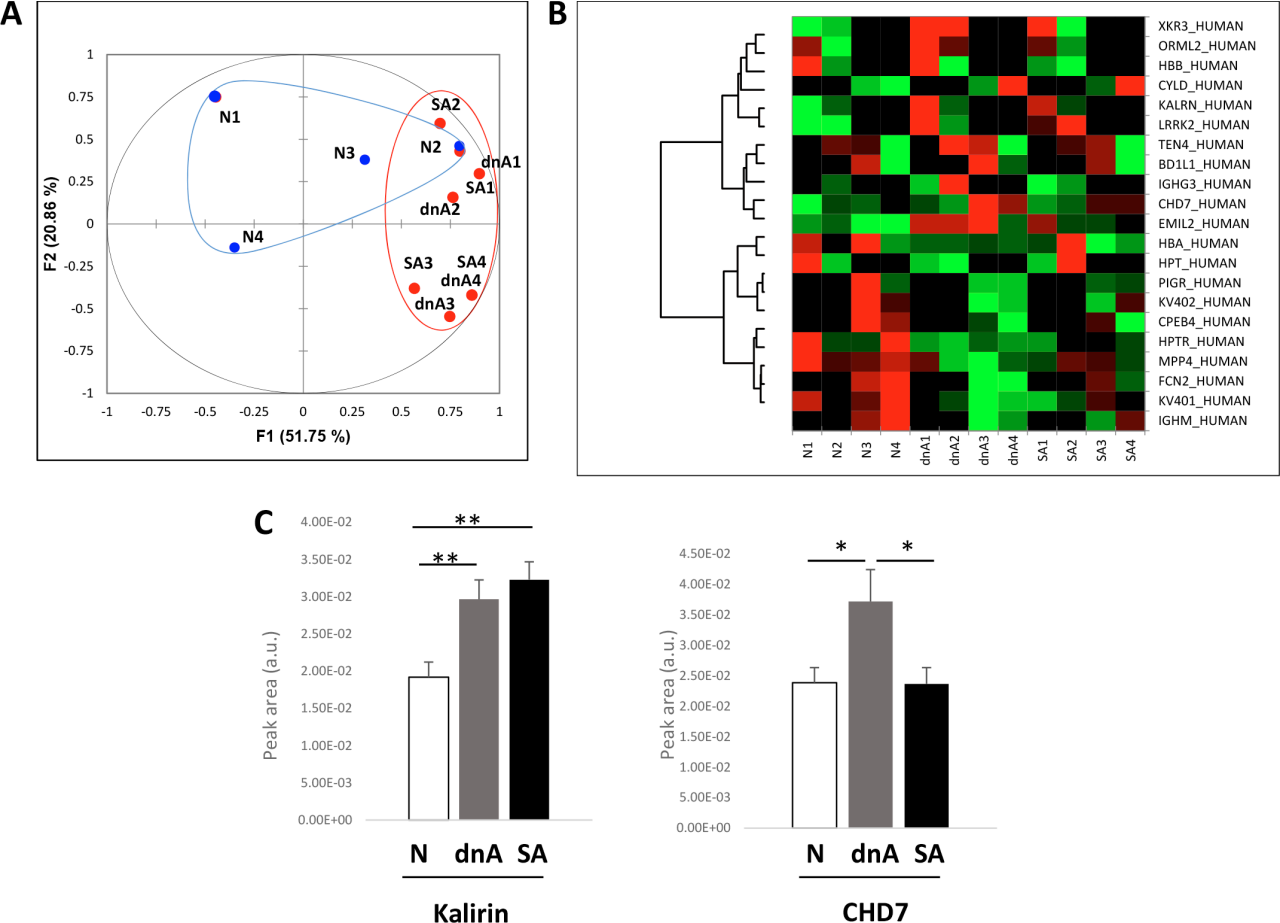
EVs from hypertensive patients with albuminuria exhibit increased levels of kalirin and CHD7 (**A**) differential analysis of EVs within N, dnA, and SA groups was performed by iTRAQ and LC-MS/MS. Proteins with log2 of Fold-change (Zq) values ± 1.5 (Fold-change = 3) were considered differentially expressed. PCA was performed using these differential proteins. The first principal component greatly separates normoalbuminuric patients from the albuminuric patients, showing the EVs from normoalbuminuric patients express very different levels of proteins to those of the albuminuric. (**B**) Heatmap showing relative quantifications of the significant proteins. (**C**) Quantification of kalirin and CHD7 was performed by SRM in an independent cohort of 99 patients: 49 albuminuric (25 dnA, 24 SA) and 50 normoalbuminuric. The increase observed in albuminuric patients in the discovery phase was confirmed for both proteins. Values are expressed as mean ± SEM.

Differential abundance analysis was performed by means of iTRAQ labelling and LC-MS/MS. To focus in the alterations occurring with albuminuria onset, two different groups attending to albuminuria development were independently analysed: a) patients developing de novo albuminuria during follow-up (dnA); b) patients with sustained albuminuria during follow-up (SA) and a nomoalbuminuric group was used as control (N). Table 1 Results showed 20 proteins significantly altered, among which 19 were found in any of the albuminuric groups, compared to the normoalbuminuric (Supplementary Table S1). A principal component analysis (PCA) and heatmap (Figure 2A and 2B) were performed using these differential proteins. In the PCA (Figure 2A) the first principal component greatly separates normoalbuminuric patients from the albuminuric patients, showing that the EVs from normoalbuminuric patients express very different levels of proteins to those of the albuminuric. All proteins were searched in two EVs databases: Vesiclepedia and EVpedia, for prior evidence of expression by EVs, either MVs or exosomes. Fourteen of them have been previously reported to be expressed by EVs at the protein level, while the mRNA of another 4 (MAGUK p55 subfamily member 4, MPP4; ORM1-like protein 2, ORML2; CHD7; and XK-related protein 3) was shown to be carried by these vesicles (Supplementary Table S1). Two proteins have never been associated with EVs before (Ubiquitin carboxyl-terminal hydrolase, CYLD; and biorientation of chromosomes in cell division protein 1-like 1). Thus, we provide new evidence of the expression of 6 proteins in EVs. Results from all quantified proteins in EVs are shown in Supplementary Table S2.

Table 1Baseline characteristics and medications of the patients recruited for discovery and confirmation phase

**Table:** 

Discovery phase				
	*N* (*n* = 8)	dnA (*n* = 7)	SA (*n* = 7)	*P*-value
**Age (years)**	62 ± 6	63 ± 11	62 ± 10	0.79
**Sex (male), %**	50	70	70	0.6
**BMI (kg/m2)**	30 ± 4	32 ± 4	30 ± 5	0.96
**Current smoking, %**	0	0	30	0.095
**Total cholesterol (mg/dl)**	194 ± 27	159 ± 16	182 ± 43	0.046
**Triglycerides (mg/dl)**	86 ± 22	97 ± 30	134 ± 77	0.13
**HDL cholesterol (mg/dl)**	56 ± 14	48 ± 8	47 ± 11	0.19
**LDL cholesterol (mg/dl)**	121 ± 28	92 ± 15	108 ± 31	0.04
**Glycaemic (mg/dl)**	100 ± 15	101 ± 16	106 ± 31	0.54
**Uric acid (mg/dl)**	4.6 ± 1.1	6 ± 2	6 ± 2	0.18
**Creatinine clearance rate (mg/ml)**	88 ± 22	114 ± 50	84 ± 43	0.48
**eGFR (ml/min/1.73m2)**	83 ± 13	68 ± 23	67 ± 28	0.29
**Systolic blood pressure (mmHg)**	126 ± 9	136 ± 24	124 ± 13	0.95
**Diastolic blood pressure (mmHg)**	75 ± 10	81 ± 4	75 ± 11	0.93

**Table:** 

Medications								
**Antihypertensives, %**				
** ACEi**	12	12	0	0.58
** ARB**	88	88	100	0.58
** Diuretic**	88	57	72	0.74
** Calcium channel blocker**	75	63	15	0.13
** Beta blocking agent**	25	29	29	0.98
** Alpha blocking agent**	13	14	14	0.3
**Other treatments, %**				
**Anticoagulant agent**	0	29	29	0.24
**Lipid lowering agents**		88		72		57				0.41		
**Antidiabetic agent**		0		14		0				0.32

**Table:** 

Confirmation phase								
	*N* (*n* = 50)	dnA (*n* = 25)	SA (*n* = 24)	*P*-value
**Age (years)**	65 ± 11	68 ± 8	64 ± 12	0.23
**Sex (male), %**	38	64	67	0.024
**BMI (kg/m2)**	30 ± 4	29 ± 5	30 ± 4	0.6
**Current smoking, %**	10	20	13	0.48
**Total cholesterol (mg/dl)**	186 ± 29	165 ± 26	174 ± 28	0.007
**Triglycerides (mg/dl)**	123 ± 51	140 ± 66	141 ± 71	0.38
**HDL cholesterol (mg/dl)**	54 ± 12	51 ± 13	47 ± 14	0.06
**LDL cholesterol (mg/dl)**	107 ± 28	86 ± 17	101 ± 21	0.002
**Glycaemic (mg/dl)**	120 ± 41	128 ± 26	118 ± 34	0.57
**Uric acid (mg/dl)**	5 ± 2	6 ± 2	7 ± 2	0.0003
**Creatinine clearance rate (mg/ml)**	99 ± 41	86 ± 46	75 ± 37	0.017
**eGFR (ml/min/1.73m2)**	80 ± 17	70 ± 22	67 ± 27	0.086
**Systolic blood pressure (mmHg)**	138 ± 17	136 ± 17	142 ± 29	0.64
**Diastolic blood pressure (mmHg)**	81 ± 11	81 ± 11	84 ± 15	0.69

**Table:** 

Medications								
**Antihypertensives, %**				
** ACEi**	14	16	20	0.80
** ARB**	86	84	80	0.80
** Diuretic**	46	60	67	0.2
** Calcium channel blocker**	64	60	75	0.51
** Beta blocking agent**	26	32	30	0.86
** Alpha blocking agent**	18	32	17	0.31
**Other treatments, %**				
** Anticoagulant agent**	40	48	25	0.24
** Lipid lowering agents**	74	76	84	0.67
** Antidiabetic agent**	28	46	28	0.36

Values are expressed as mean ± SD or percentages (%). *P*-value was calculated using one-way ANOVA. BMI: body mass index; HDL: high-density lipoprotein cholesterol; LDL: low-density lipoprotein cholesterol; eGFR: estimated glomerular filtration rate. N: normoalbuminuria; dnA: *de novo* albuminuria; SA: sustained albuminuria.

### Confirmation by SRM of the increased abundance of kalirin and CHD7 in EVs from hypertensive patients with albuminuria and analysis of correlation with E-selectin

Kalirin and CHD7 were analysed by SRM together with the cellular marker of activated ECs, E-selectin (CD62E). Quantification of these proteins was performed in an independent cohort of 99 patients: 49 albuminuric (25 dnA, 24 SA) and 50 normoalbuminuric. Both kalirin and CHD7 were found to significantly increase in dnA (Figure 2C and Table 2). The former was increased in SA in a similar way, while levels of CHD7 in SA were similar to those of normoalbuminuric patients. A significant positive correlation of both proteins with CD62E was found in EVs (Table 2).

### *In vitro* analysis of the expression of kalirin and CHD7 in TNF-α treated ECFCs

**Figure 3:**
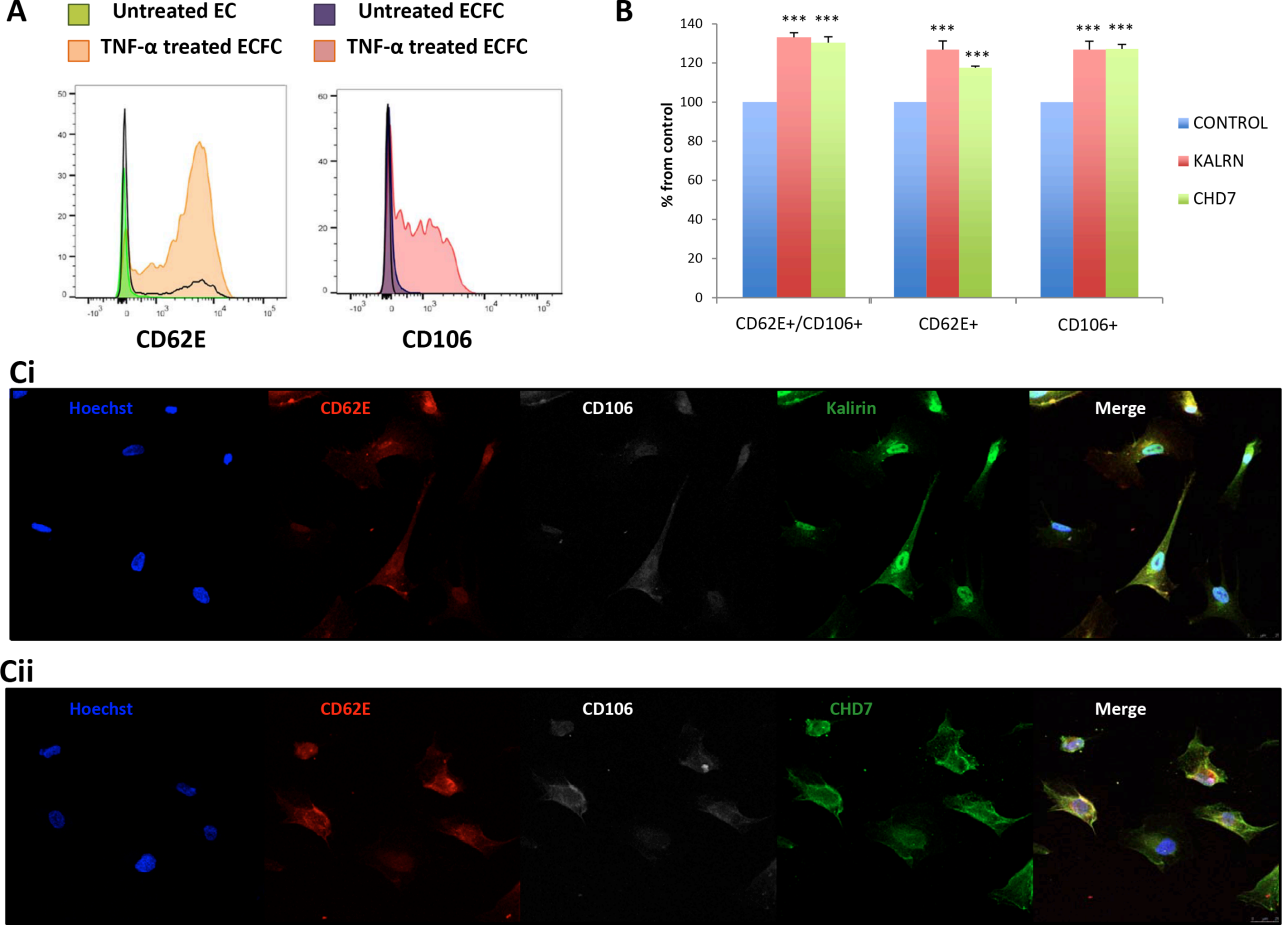
Kalirin and CHD7 are increased in endothelial cells upon activation Cells were treated with TNF-α or EC-medium alone for 5 h. (**A**, **B**) After activation, cells were trypsinized and labelled 1 h with the surface antibodies CD62E-APC and CD106-PE. After this, cells were fixed using 4% paraformaldehyde and permeabilized before incubating 90 min at 4°C with primary antibodies for the intracellular proteins kalirin and CHD7. All samples were analysed in a FACS CANTO II (BD Biosciences). A, Flow cytometry of TNF-α shows increased expression of the activation markers CD62E and CD106. B, Analysis of TNF-α activated cells by flow cytometry proved a significant increase of both kalirin and CHD7 in cells expressing CD62E and/or CD106. Control cells for this experiment were CD62^-^/CD106^-^. (**C**) Cells were fixed using 4% paraformaldehyde, permeabilized and labelled with CD62E-APC and CD106-PE as well as with either of the primary antibodies for kalirin or CHD7. After this, coverslips were incubated with a donkey anti-rabbit secondary antibody conjugated with Alexa-488 and Hoechst dye. Cells were visualized in a TCS SP5 confocal microscope (Leica). Immunofluorescence analysis of TNF-α treated cells confirmed the increase of kalirin (**C1**) and CHD7 (**C2**) in cells expressing any or both of the activation markers. All experiments were performed with at least *n* = 3. Values are expressed as mean ± SEM. KALRN: kalirin, E-selectin = CD62E, VCAM-1 = CD106.

**Figure 4:**
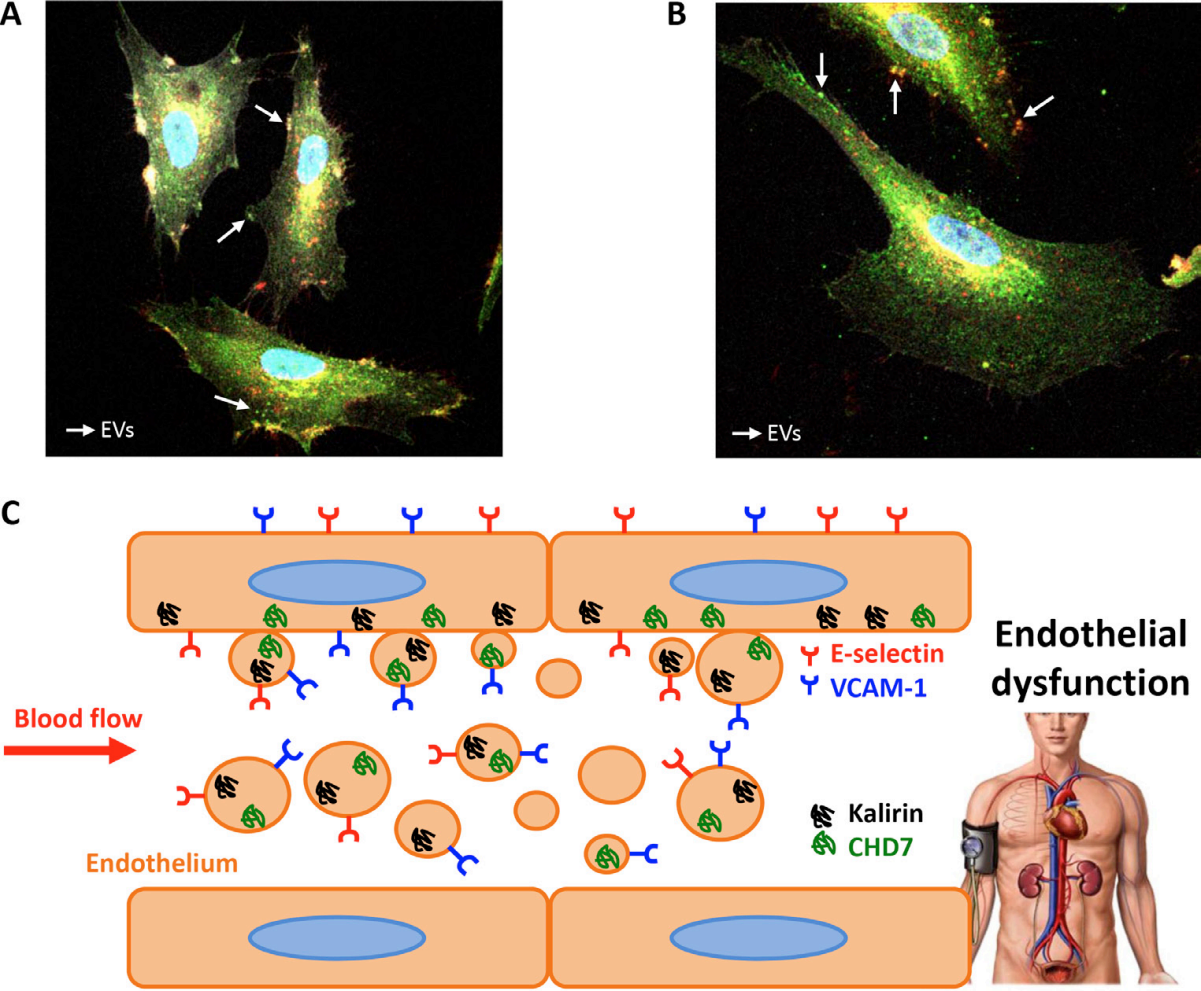
EVs from endothelial cells express kalirin and CHD7 Immunofluorescence analysis of kalirin and CHD7 (green), CD62E (red) and CD106 (white) of TNF-α activated cells showed proved the expression of kalirin (**A**) and CHD7 (**B**) proteins by EVs. (**C**) Schematic conclusion. Overexpression of Kalirin and CHD7 proteins by activated endothelial cells due to increased shear stress may result in the observed increase in circulating EVs of hypertensive patients with albuminuria.

In order to test the hypothesis that kalirin and CHD7 would increase in blood vessel ECs upon activation, ECs were isolated from human saphenous vein. These primary cultures provided us with an *in vitro* model of adult blood vessel ECs, which might be more reliable than commonly used human umbilical vein ECs (HUVECs). Expression of CD62E and CD106 was substantially increased in TNF-α treated cells compared to untreated controls as shown by flow cytometry (Figure 3A). Flow cytometric analysis of kalirin and CHD7 in TNF-α treated cells showed a significant increase in the expression of both proteins (*p* < 0.001) in all activated cells (CD62E^+^, CD106^+^ and CD62E^+^/CD106^+^) as compared to the double negatives (CD62E^-^/CD106^−^) (Figure 3B). Immunocytofluorescence analysis of kalirin and CHD7, together with both activation markers showed similar results to those observed by flow cytometry and proved expression of the two proteins in ECs (Figure 3C) as well as in EVs budding from TNF- α activated cells (Figure 4A and 4B).

## DISCUSSION

The development of albuminuria during RAS suppression involves increased shear stress [11], which triggers endothelial activation and subsequent dysfunction [5]. Besides, an increased oxidative stress has been shown to occur in these patients [6]. Reactive oxygen species (ROS) decrease endothelial nitric oxide (NO) bioavailability [12], which also results in endothelial dysfunction. On the other hand, endothelial activation occurring in the kidney from hypertensive patients leads to endothelial damage which reduces albumin filtration and provokes albuminuria [13]. For all these reasons, new indicators of albuminuria progression are needed to better characterize vascular condition of hypertensive patients. In this sense, analysis of proteins carried by circulating EVs seems a very interesting approach, since shear stress enhances release of EEVs [14] resulting in increased levels in the bloodstream of albuminuric patients [10].

**Table 2:** Results from the SRM confirmation analysis

Protein	Alter.	ANOVA	Ratio dNA_N	Tukey	Ratio SA_N	Tukey	Ratio SA_dnA	Tukey	CD62E Correl.
Kalirin	↑dnA_N ↑SA_N	0.0007	1.55	0.0064	1.68	0.0014	1.09	NS	p = 5.96E^-11^ R^2^ = 0.59
CHD7	↑dnA_N ↓SA_N	0.0193	1.56	0.0282	0.99	NS	0.64	0.0495	p = 3.02E^-08^ R^2^ = 0.52

Using a proteomics approach, we found 2 proteins, kalirin and CHD7, increased in patients developing dnA, which therefore may be indicators of the appearance of albuminuria in hypertensive patients under RAS blockage. Besides, a positive correlation of kalirin and CHD7 with the endothelial activation marker CD62E was also found. This result suggested these 2 proteins may be released by endothelial cells upon activation via EVs. We could confirm a significant increase in levels of kalirin and CHD7 in activated ECs *in vitro*. This proves an involvement of both proteins in endothelial cell activation within blood vessels and explains the observed increase in EVs from albuminuric patients, considering the inherent endothelial dysfunction associated. The results obtained *in vitro* showed a similar increase of CHD7 and kalirin upon EC activation, but a recovery in CHD7 up to normal levels was found in SA patients, which point to a different behaviour of both proteins. CHD7 seems to be an acute marker of endothelial dysfunction, which explains the increase in de novo albuminuria and the recovery in sustained albuminuria. On the other hand, kalirin seems to be a more general indicator of endothelial damage as increases in both albuminuric groups.

Kalirin, also called Duo, is involved in signal transduction. Its gene has been associated with susceptibility of coronary artery disease [15] and ischemic stroke [16]. Its expression by VSMCs has been associated with neointimal hyperplasia in mice [17]. Here we provide, for the first time, evidence of the expression of kalirin in human ECs and of its involvement in processes leading to endothelial dysfunction. Kalirin is known to inhibit iNOS [17, 18] and the hypothesis that it may also suppress NO availability in the endothelium by inhibiting eNOS is reasonable and deserves further research. Interestingly, NO release by ECs has been shown to decrease the release of EEVs [14] and therefore a potential inhibition of NO production triggered by kalirin is in accordance with both the observed augment in activated ECs and circulating EVs from albuminuric patients.

There is not much information to date about protein CHD7 which functions as a transcriptional activator [19]. It is crucial during embryogenesis and has been involved in heart and great vessel development [19, 20]. Mutations in *Chd7* gene are responsible for CHARGE syndrome, in which, heart and vascular system are impaired [21]. Therefore, CHD7 is deeply involved in cardiovascular system development and its correct function may be important for a good cardiovascular condition. In our work, increased expression of CHD7 by EVs has been linked to albuminuria and endothelial activation. Indeed, to date, there was no evidence of the expression of CHD7 by ECs, which we hereby have proved by both flow cytometry and immunofluorescence.

A limitation of our study might be the lack of sex matching among populations in the confirmation phase. We have anyway taken into consideration the existing differences in the correlation of albuminuria levels and disease state by adjusting albuminuria ranges according to gender, as a way of correcting this limitation. Further studies in greater cohorts will allow to assess potential of these biomarkers and evaluate influence of gender.

The hereby presented results support a potential involvement of kalirin and CHD7 proteins in human blood vessel endothelial dysfunction and show an increased expression of both by circulating EVs in albuminuric patients (Figure 4C). Hence, we propose levels of these proteins in circulating EVs as novel endothelial dysfunction markers to monitor vascular condition in hypertensive patients with albuminuria.

## MATERIALS AND METHODS

For more details of the experimental procedures, please see *Supplementary material*.

### Patients’ recruitment

Patient selection and classification was previously described [1–4]. Briefly, 121 patients hypertensive patients with (*n* = 63) or without albuminuria (*n* = 58) were recruited between January 2012 and June 2013 after being followed for a minimum period of 3 years with visits to the Hypertension Unit, Hospital Universitario 12 de Octubre, Madrid, Spain; at least, every 6 months. After that, the patients continued with their annual revisions. Patients were classified according to high albuminuria development during follow-up in 3 groups: (a) patients with persistent normoalbuminuria (*N*, *n* = 58); (b) patients developing de novo albuminuria during follow-up (dnA, *n* = 32); (c) patients with sustained albuminuria during follow-up (SA, *n* = 31). Characteristics and medications of the specific cohorts recruited for the discovery and confirmation phase are given in Table 1 A human saphenous vein from revascularization surgery at Hospital Virgen de la Salud, Toledo, Spain; was collected for isolation of the ECs addressed for primary cultures.

The study was conducted according to recommendations of Declaration of Helsinki and approved by the local Ethics Committee. Informed consent was requested from subjects prior to inclusion in the study.

### Extracellular vesicles (EVs) isolation

EVs were isolated as previously described [22], with minor modifications. For more details see *Supplementary material*.

### Electron microscopy (EM)

The precipitate of EVs obtained by ultracentrifugation was fixed in paraformaldehyde 4% and visualized after negative staining in a JEM 1010 (JEOL Peabody, MA, USA).

### iTRAQ labelling

To focus in the alterations occurring with albuminuria onset, two different groups attending to albuminuria development were independently analysed (dnA and SA, *n* = 14, 7 of each group). A nomoalbuminuric group (*n* = 8) was used as control. In order to analyse four samples per group, EVs from 2 patients were pooled when necessary. Two different 8-plex experiments were performed to process all the analysed sample pools. In every experiment two reference samples, each composed of a pool of 4 samples obtained from healthy blood donors, were labelled with the tags 113 and 117, in order to normalize quantifications and secure accurate comparison of samples between experiments. The tags addressed to label every sample are available on Supplementary Table S3.

### LC-MS/MS and differential analysis

Labelled peptide samples were analysed by LC-MS/MS in a Q-Exactive mass spectrometer (Thermo Fisher Scientific). Protein quantification from reporter ion intensities and statistical analysis were performed using QuiXoT software. All the significant proteins found in the differential analysis were searched against two EVs databases: Vesiclepedia and EVpedia in order to find previous evidence of their expression in EVs, both MVs and exosomes, at the protein or mRNA level.

### SRM confirmation

For confirmation, EVs from an independent cohort of 99 patients were analysed by SRM: 49 albuminuric (25 dnA, 24 SA) and 50 normoalbuminuric. SRM transitions (3 per peptide) were monitored during an individual sample analysis (SRM peptides analysed and settings are shown in Supplementary Table S4 and chromatograms in Supplementary Figure S2).

### Isolation and TNF-α activation of ECs

ECs were isolated from human saphenous vein and grown as previously described [23]. Endothelial phenotype of ECs was checked by flow cytometry and confocal microscopy (Supplementary Figure S1). For stimulating ECs, cells were seeded at a density of 2.5 × 10^4^ cells/cm^2^, treated with 10 ng/ml tumour necrosis factor- α (TNF-α) or EC-medium alone for 5 h.

### Flow cytometry

EVs were analysed by flow cytometry using platelet marker CD61 (most abundant source of EVs in blood). EVs gate was defined using MegaMix SSC Plus beads (Biocytex).

For analysing ECs, cells were trypsinized after stimulation and labelled with the surface antibodies for E-selectin: CD62E-APC; and VCAM-1: CD106-PE as well as with either of the intracellular proteins kalirin and CHD7; followed by a donkey anti-rabbit secondary antibody conjugated with Alexa-488.

All samples were analysed in a FACS CANTO II (BD Biosciences). Experiment was repeated 3 times.

### Confocal microscopy

For visualizing EVs staining of CD61 or its isotype control was assayed. For ECs analysis, cells were seeded in coverslips and treated with TNF-α or EC-medium alone. Detection of EC activation markers E-selectin and VCAM-1 was accomplished, together with either of the intracellular staining of kalirin or CHD7 using the same antibodies employed for flow cytometry.

EVs and cells were visualized in a TCS SP5 confocal microscope (Leica). All experiments were performed with *n* = 3.

### Statistical analysis

Values for patients’ characteristics are expressed as means ± standard deviation (SD) or percentages. One-way ANOVA was used to calculate statistically significant differences of the values between different groups. Post-hoc analysis of significant ANOVA results was performed by means of Tukey analysis. In iTRAQ results, we have considered differentially expressed those proteins with log2 of Fold-change (Zq) values ± 1.5 (Fold-change = 3). For *in vitro* analyses, Student's *T*-test was performed and each experiment was repeated 3 times.

All statistics were calculated using SPSS 15.0 software, except for PCA and heatmap, which were done with XLSTAT for Excel (Microsoft).

## SUPPLEMENTARY MATERIALS FIGURES AND TABLES




