# Supplementary material for: Kalirin and CHD7: novel endothelial dysfunction indicators in circulating extracellular vesicles from hypertensive patients with albuminuria
Source: Oncotarget. 2017 Feb 1;8(9):15553–62. doi: 10.18632/oncotarget.14948 (PMC5362505; doi:10.18632/oncotarget.14948)
Supplement: Supplementary file 1 [file oncotarget-08-15553-s001.pdf]

Kalirin and CHD7: novel endothelial dysfunction indicators in circulating extracellular vesicles from hypertensive patients with albuminuria

Supplementary Materials

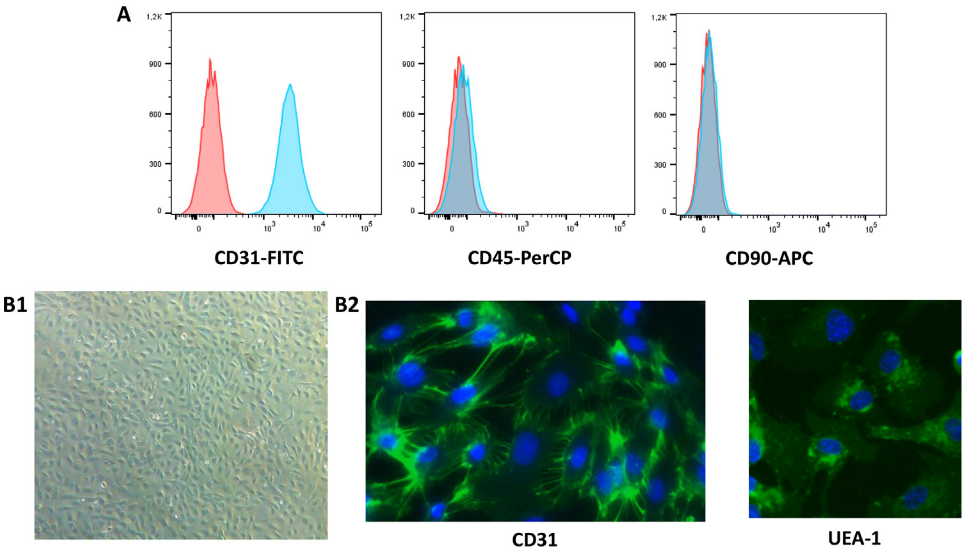

Supplementary Figure S1: Endothelial cell phenotype check of human saphenous vein ECs. (**A**) Endothelial cells isolated from human saphenous vein were analysed by flow cytometry, and were found to be $CD31^{+}$ (endothelial marker), $CD45^{-}$ (hematopoietic marker) and $CD90^{-}$ (mesenchymal marker, used to rule out contamination of mesenchymal cells). (**B1**), Bright field image of ECs. (**B2**), Immunocytofluorescence analysis showing positivity for endothelial markers CD31 and lectin UEA-1.

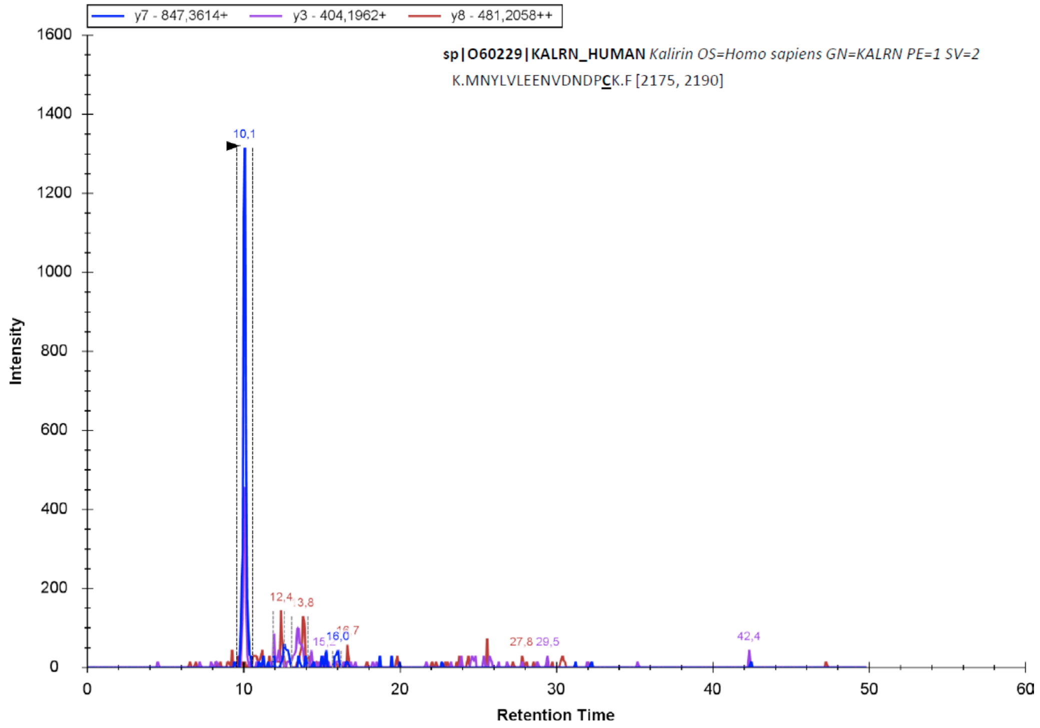

Supplementary Figure S2: Chromatograms from peptides and transitions used for SRM analysis. SRM analysis was carried out in a TEMPO nano LC system coupled to a 4000 QTRAP modified triple quadrupole (AB Sciex). The 3 most intense transitions from each peptide were selected for the quantification.

Supplementary Table S1: Experimental design used for iTRAQ analysis

	113	114	115	116	117	118	119	121
**Experiment 1**	C1	N1	dnA1	SA1	C2	N2	dnA2	SA2
**Experiment 2**	C1	dnA3	SA3	N3	C2	dnA4	SA4	N4

Two different groups attending to albuminuria development were independently analysed (dnA and SA, n = 14, 7 of each group). A nomoalbuminuric group (n = 8) was used as control. Two different 8-plex iTRAQ experiments were performed to analyze all samples and EVs from 2 patients were pooled when necessary. The 4 samples analyzed per group were labelled with a different tag to avoid labelling bias. A pool of 4 samples obtained from healthy blood donors, was labelled with the tags 113 and 117, in order to normalize quantifications and secure accurate comparison of samples between experiments.

Supplementary Table S2: Peptides, transitions and settings used for SRM quantification

Protein Name	Protein reference Uniprot, gen name)	Peptide Sequence	Precursor	Product Mz	Transition	Declustering Potential	Collision Energy	Dwell time
Kalirin	sp|O60229|KALRN_HUMAN	MNYLVLEENVDNDPCK	651,6291+++	847.361444	V - y7+	78.6	27.3	70
			651,6291+++	404.196216	P - y3+	78.6	27.3	70
			651,6291+++	481.205824	N - y8++	78.6	27.3	70
Chromodomain-helicase-DNA-binding protein 7	sp|Q9P2D1|CHD7_HUMAN	WTDILSHGR	542,7803++	456.231356	S - y4+	70.7	26.7	60
			542,7803++	341.70338	I - y6++	70.7	26.7	60
			542,7803++	285.161348	L - y5++	70.7	26.7	60
E-selectin	sp|P16581|LYAM2_HUMAN	DEDCVEIYIK	642,2948++	924.485916	C - y7+	77.9	32.3	60
			642,2948++	462.746596	C - y7++	77.9	32.3	60
			642,2948++	360.103755	D - b3+	77.9	32.3	60
		GYMNCLPSASGSFR	773,8425++	1081.509505	C - y10+	87.5	39.8	60
			773,8425++	356.174652	S - y7++	87.5	39.8	60
			773,8425++	352.132553	M - b3+	87.5	39.8	60

SRM analysis was carried out in a TEMPO nano LC system coupled to a 4000 QTRAP modified triple quadrupole (AB Sciex). All quantified peptides met a stringent criteria in which at least 6 transitions of the peptide were shown to co-elute in the chromatogram. Among these, the 3 most intense transitions were selected for the quantification. Besides, all peptides used for SRM quantification were searched against SRM Atlas database (http://www.mrmatlas.org/index.php). All had been previously used for SRM quantification of our proteins of interest and detected in similar conditions in a 4000 QTRAP mass spectrometer.

Supplementary Table S3: Differential proteins found in the iTRAQ analysis of EVs

Gene	Protein	Alter.	Zq dNA_N	Zq SA_N	Zq SA_dnA	EVs DB
MPP4_HUMAN	MAGUK p55 subfamily member 4. MPP4	↓dnA ↓SA	−3.59	−1.64	1.94	MV; mRNA
HPT_HUMAN	Haptoglobin	↓dnA ↑SA	−2.17		2.79	MV. E; Protein
HPTR_HUMAN	Haptoglobin-related protein	↓dnA	−1.59			MV. E; Protein
KV402_HUMAN	Ig kappa chain V-IV region Len	↓dnA ↓SA	−2.73	−1.88		MV; Protein
KV401_HUMAN	Ig kappa chain V-IV region (Fragment)		−2.06			MV; Protein
CPEB4_HUMAN	Cytoplasmic polyadenylation element-binding protein 4	↓dnA ↓SA	−1.74	−1.75		MV; Protein
FCN2_HUMAN	Ficolin-2	↓dnA	−1.55			MV. E; Protein
HBB_HUMAN	Hemoglobin subunit beta	↓SA		−2.88	−1.72	MV. E; Protein
IGHG3_HUMAN	Ig gamma-3 chain C region	↓SA			−2.29	MV. E; Protein
LRRK2_HUMAN	Leucine-rich repeat serine/threonine-protein kinase 2	↑dnA ↑SA	1.68	2.15		MV. E; Protein
KALRN_HUMAN	Kalirin	↑dnA	1.88			MV. E; Protein
ORML2_HUMAN	ORM1-like protein 2. ORML2	↑dnA	2			MV; mRNA
EMIL2_HUMAN	EMILIN-2	↑dnA	2.17			MV; Protein
CHD7_HUMAN	Chromodomain-helicase-DNA-binding protein 7. CHD7	↑dnA ↓SA	2.62			MV. mRNA
CYLD_HUMAN	Ubiquitin carboxyl-terminal hydrolase. CYLD	↑dnA ↑SA	2.63	2.61		NO
BD1L1_HUMAN	Biorientation of chromosomes in cell division protein 1-like 1	↓SA			−1.86	NO
PIGR_HUMAN	Polymeric immunoglobulin receptor	↓dnA ↓SA	−2.05	−1.6		MV. E; Protein
IGHM_HUMAN	Ig mu chain C region	↓dnA	−1.91			MV. E; Protein
XKR3_HUMAN	XK-related protein 3	↑dnA	1.57			MV; mRNA
TEN4_HUMAN	Teneurin-4	↑dnA ↓SA	2.8		−1.63	MV. E; Protein

Alter: alteration. EVs DB: previous evidence of expression and molecular level (protein or mRNA) in EVs according to EVpedia and Vesiclepedia databases. MV: microvesicles. E: exosomes.

Supplementary Table S4: Results iTRAQ LC-MS/MS. See Supplementary_Table_S4

EXPERIMENTAL PROCEDURES

Extracellular vesicles (EVs) isolation

An amount of 14 ml of blood was collected from each patient using sodium citrate tubes. Platelet-free plasma (PFP) was obtained by 2 centrifugations: 1500·g, 25 min and 15,000·g, 2 min. Then, isolation of EVs by ultracentrifugation. A series of 3 ultracentrifugation steps were carried out at 250,000·g in a SW41 rotor of an Optima L-100 XP Ultracentrifuge (Beckman Coulter): 1) HEPES 5 h 2) KBr 250 mM 15 h and 3) PBS 4 h; with the goal in mind of isolating EVs without contamination of the proteins from blood plasma. All of these were performed with the addition of 10 mM sodium citrate to prevent aggregation of EVs.

Electron microscopy (EM)

The precipitate of EVs obtained by ultracentrifugation was suspended in 1 ml paraformaldehyde 4% in PBS. A 15 µl droplet of sample was placed in an electron microscopy Formvar/carbón grid (Ted Pella) for 10 min. After 5 min in bidistilled water, a negative staining was carried out using 20 µl of uranile acetate 2%, 30 sec. The sample was visualized in a JEM 1010 (JEOL Peabody, MA, USA).

iTRAQ labelling

To focus in the alterations occurring with albuminuria onset, two different groups attending to albuminuria development were independently analysed (dnA and SA, $n = 14$, 7 of each group). A nomoalbuminuric group ($n = 8$) was used as control. Isolated EVs from ultracentrifugation were lysed using 500 µl of lysis buffer (urea 7M, tiourea 2M, CHAPS 4%, SDS 1%, DTT 1%) and two 2 min sonication steps. After centrifugation at 15,000·g, 5 min, supernatant was collected and quantified using the Bradford-Lowry method. In order to analyse four samples per group, EVs from 2 patients were pooled when necessary. For each analysed sample, a 150 µg aliquot was precipitated with cold acetone and suspended in Tris 50 mM, SDS 4%, DTT 50 mM adjusted to pH 8 and then loaded into PAGE-SDS gels to concentrate all the proteins in a single band. Proteins were in-gel digested overnight at 37°C with 60 ng/ml modified trypsin (Promega) at 12:1 protein:trypsin (w/w) ratio in 50 mM ammonium bicarbonate, pH 8.8 containing 10% acetonitrile. For stable isobaric labelling, the resulting tryptic peptides were dissolved in triethylammonium bicarbonate (TEAB) buffer and labelled using two 8-plex iTRAQ Reagents Multiplex Kits (AB Sciex) according to manufacturer›s protocol. Samples were labelled with iTRAQ reagents at room temperature (RT) for 1 h previously reconstituted with 70 µl of isopropanol. Reaction was stopped after incubation at RT for 2 h with 0,5% trifluoroacetic acid (TFA) and peptides were combined. Two different 8-plex experiments were performed to process all the analysed sample pools. In every experiment two reference samples, each composed of a pool of 4 samples obtained from healthy blood donors, were labelled with the tags 113 and 117, in order to normalize quantifications and secure accurate comparison of samples between experiments. The tags addressed to label every sample are available on Supplementary Table S1.

LC-MS/MS and differential analysis

Labelled peptide samples were analysed by LC-MS/MS using a C-18 reversed phase nano-column (75 µm I.D × 50 cm, 2 µm particle size, Acclaim PepMap RSLC, 100 C18 (Thermo Fisher Scientific) in a continuous acetonitrile gradient consisting of 0–30% B in 360 min, 50–90% A in 3 min (A = 0.5% formic acid; B = 90% acetonitrile, 0.5% formic acid). A flow rate of 200 nL/min was used to elute peptides from the reverse phase nano-column to an emitter nanospray needle for real time ionization and peptide fragmentation in a Q-Exactive mass spectrometer (Thermo Fisher Scientific). For increasing proteome coverage, iTRAQ-labelled samples were also fractionated by cation exchange chromatography (Oasis HLB-MCX columns) into six fractions, which were desalted and analysed by using the same system and conditions described before.

Spectra analysis for peptide identification was performed with the program Proteome Discoverer version 1.4.0.29 using SEQUEST-HT (Thermo Fisher Scientific). Only peptides with a confidence of at least 95% were used to quantify the relative abundance of each peptide determined. Protein quantification from reporter ion intensities and statistical analysis were performed using QuiXoT software. In this model protein log2-ratios are expressed in form of the standardized variables, i.e., in units of standard deviation according to their estimated variances (Zq values). Cut-off for signification was set at $Zq = \pm 1.5$. All the significant proteins found in the differential analysis were searched against two EVs databases: Vesiclepedia and EVpedia in order to find previous evidence of their expression in EVs, both MVs and exosomes, at the protein or mRNA level.

SRM confirmation

For confirmation, an independent cohort of 99 patients was recruited: 49 albuminuric (25 dnA, 24 SA) and 50 normoalbuminuric. Samples were mixed according to their albuminuric levels in 34 pools prior to analysis. Protein samples were reduced with DTT 100 mM (Sigma Aldrich) and alkylated with iodoacetamide 550 mM (Sigma Aldrich), both in ammonium bicarbonate 50 mM (99% purity; Scharlau). The proteins were digested in ammonium bicarbonate 50 mM, acetonitrile 15% (LC-MS grade, Scharlau) with sequencing grade modified porcine trypsin (Promega) at a final concentration of 1:50. After digestion at 37°C overnight, 2% formic acid (99.5% purity; Sigma Aldrich) was added and samples were

cleaned with Pep-Clean spin columns (Pierce), according to manufacturer's instructions. Tryptic digests were dried in speed-vac and resuspended in 2% acetonitrile, 2% formic acid prior to MS analysis The LC-MS/MS system consisted of a TEMPO nano LC system coupled to a 4000 QTRAP modified triple quadrupole (AB Sciex). Three replicate injections (4 µL containing 20 µg of protein) were performed per sample. RPLC was achieved on a C18 column (Onyx Monolithic C18, 150 × 0.1mm I.D., Phenomenex). SRM transitions (3 per peptide) were monitored during an individual sample analysis (SRM peptides analysed and settings are shown in Supplementary Table S2). IntelliQuan algorithm included in Analyst 1.4.5 software was used to calculate abundances on the basis of peak areas after integration and normalization with the total ion chromatogram (TIC). All quantified peptides met a stringent criteria in which at least 6 transitions of the peptide were shown to co-elute in the chromatogram, therefore securing unambiguous assignation of the selected peptides to the corresponding protein. Among these, the 3 most intense transitions were selected for the quantification (Supplementary Table S2). Besides, all peptides used for SRM quantification were searched against SRM Atlas database (http://www.mrmatlas.org/index.php). All had been previously used for SRM quantification of our proteins of interest and detected in similar conditions in a 4000 QTRAP mass spectrometer.

Isolation of ECs

After clamping the branches from the human saphenous vein, it was washed with PBS and infused with tripsin-EDTA 0.1% for 5 min. Then, the vessel was perfused with D10 medium (Dulbecco's Modified Eagle Medium, DMEM, 10% fetal bovine serum, FBS, 1 × L-Glutamine–Penicillin–Streptomycin, GPS) and the media collected, centrifuged at 300·g and the pelleted cells suspended in EC-medium (Endothelial Cell Growth Medium-2, EGM-2, except for hydrocortisone; Lonza, 20% FBS, 1x GPS). EC were grown and obtained as previously described[1]. Endothelial lineage of ECs was checked by flow cytometry by analysing positive expression of CD31 (FITC labelled, BD Biosciences) and negative expression of CD45 (PerCP labelled, BD Biosciences) and CD90 (APC labelled, BD Biosciences) (Suppl figure 1). By means of confocal microscopy endothelial phenotype was further proved showing CD31 expression as well as binding of *Ulex europaeus* agglutinin I lectin (UEA-1; Vector Laboratories) (Supplementary Figure S1).

Flow cytometry

A 100 µl aliquot of the EVs was analysed by flow cytometry with an antibody against CD61 or its isotype control, both coupled to FITC (BD Biosciences). EVs gate was defined using MegaMix SSC Plus beads (Biocytex).

For analysing ECFCs, cells were seeded at a density of 2.5×10^4 cells/cm^2, treated with 10 ng/ml tumor necrosis factor- α (TNF-α) or EC-medium alone for 5 h, trypsinized and labelled 1 h with the surface antibodies for E-selectin: CD62E-APC; and VCAM-1: CD106-PE (BD Biosciences). After this, cells were fixed using 1% formaldehyde and permeabilized with 0.1 % triton X-100 in PBS before incubating 90 min at 4°C with either of the primary antibodies for the intracellular proteins kalirin (Santa Cruz) and CHD7 (Abcam). Then, they were incubated with a donkey anti-rabbit secondary antibody conjugated with Alexa-488 (Thermo Fisher Scientific), 90 min at 4°C.

All samples were analysed in a FACS CANTO II (BD Biosciences). All experiments were performed with at least $n = 3$.

Confocal microscopy

For visualizing EVs, two aliquots of 50 µl from the isolated EVs were suspended in 4% formaldehyde and placed in 2 coverslips and air dried. Then, they were blocked 1 h with 5% BSA and incubated 16 h with the same antibodies used for flow cytomertry. Coverslips were mounted using Mowiol 4-88 (Sigma-Aldrich).

For ECFCs analysis, cells were seeded at a density of 2.5×10^4 cells/cm^2 in coverslips and treated with TNF-α or EC-medium alone for 5 h. Then, cells were fixed using 4% paraformaldehyde, permeabilized with 0.1 % triton X-100 in PBS and incubated 16 h at 4°C with CD62E-APC and CD106-PE, as well as with either of the primary antibodies for kalirin or CHD7. After this, coverslips were incubated with a donkey anti-rabbit secondary antibody conjugated with Alexa-488 and Hoechst dye.

EVs and cells were visualized in a TCS SP5 confocal microscope (Leica). All experiments were performed with at least $n = 3$.

REFERENCES

1 Lin RZ, Moreno-Luna R, Li D, Jaminet SC, Greene AK, Melero-Martin JM. Human endothelial colony-forming cells serve as trophic mediators for mesenchymal stem cell engraftment via paracrine signaling. Proc Natl Acad Sci USA. 2014; 111:10137–10142.
